# Cobalt oxide nanoparticles can enter inside the cells by crossing plasma membranes

**DOI:** 10.1038/srep22254

**Published:** 2016-02-29

**Authors:** Elena Bossi, Daniele Zanella, Rosalba Gornati, Giovanni Bernardini

**Affiliations:** 1 Department of Biotechnology and Life Sciences, University of Insubria; Via Dunant 3, Varese, Italy; 2 Interuniversity Center “The Protein Factory”, Politecnico di Milano and Università dell’Insubria, Via Mancinelli 7, I-20131 Milan, Italy

## Abstract

The ability of nanoparticles (NPs) to be promptly uptaken by the cells makes them both dangerous and useful to human health. It was recently postulated that some NPs might cross the plasma membrane also by a non-endocytotic pathway gaining access to the cytoplasm. To this aim, after having filled mature *Xenopus* oocytes with Calcein, whose fluorescence is strongly quenched by divalent metal ions, we have exposed them to different cobalt NPs quantifying quenching as evidence of the increase of the concentration of Co^2+^ released by the NPs that entered into the cytoplasm. We demonstrated that cobalt oxide NPs, but not cobalt nor cobalt oxide NPs that were surrounded by a protein corona, can indeed cross plasma membranes.

It is well known that nanoparticles (NPs) readily enter cells^1^ by different endocytotic mechanisms^2^,^3^,^4^,^5^,^6^. The capability of NPs to be promptly uptaken by the cells, as well as that of crossing biological barriers^7^,^8^,^9^, makes them at the same time potentially dangerous and useful to human health. Dangerous, as NPs might exert their toxicity, once inside the cell, very close to target organelles as nuclei and mitochondria, a phenomenon which is referred to as Trojan horse effect^10^,^11^,^12^. Useful, as they can be directed to exert their toxicity toward cancer cells, used for drug delivery, injected as a contrast agent for diagnostic and even for theranostic purposes^13^, and assumed for food supplementation.

Recently, it has been considered the possibility that some NPs might also cross the plasma membrane by a non-endocytotic pathway^14^,^15^,^16^,^17^,^18^,^19^ gaining a direct access to the cytoplasm. This pathway is usually poorly considered as it challenges the idea of non-permeability of membranes to large hydrophilic molecules. To verify this possibility, we have set up a new protocol that has proved capable to follow the NP-plasma membrane dynamics and we have demonstrated that cobalt oxide NPs, but not cobalt nor cobalt oxide NPs that were surrounded by a protein corona, can cross plasma membranes.

Cobalt NPs have a large use in industrial and biomedical applications. They efficiently catalyse the combustion of various hydrocarbons^20^ and the degradation of water pollutants^21^ providing a cheap candidate to replace noble metals. Cobalt NPs are also used in electrocatalysis for the oxygen evolution reaction^22^, important in hydrogen generation. Recently, it has been shown that cobalt NPs can self-assemble to constitute photonic hyper-crystals^23^, which might have a strong potential in biological and chemical sensing. Moreover, cobalt NPs are magnetic and this property allows to manipulate them in a chemical or biological system using an external magnet. Moreover, magnetic NPs can be easily conjugated to biologically important constituents such as DNA, peptides, antibodies^24^ as well as enzymes^25^,^26^ and sugars^27^,^28^ to construct versatile bio-nano hybrids.

## Results and Discussion

### Calcein as “Metal Detector” in *Xenopus laevis* oocytes

**Figure 1:**
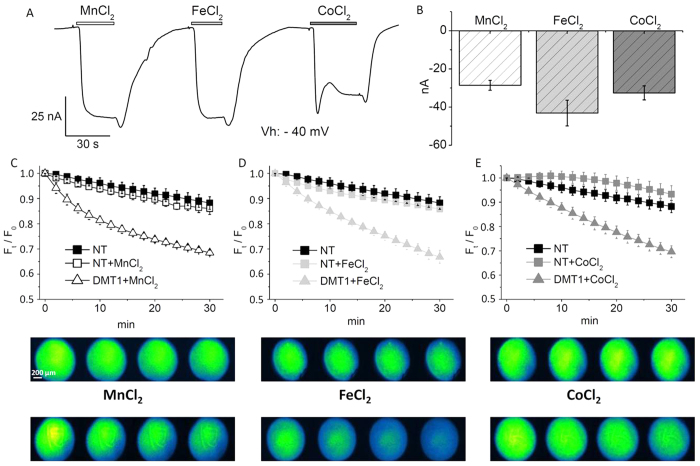
Calcein as a cytoplasmic “metal detector”. (**A**): two electrode voltage clamp of a representative rDMT1 transfected oocyte; inward currents are induced by 100 μM MnCl_2_, FeCl_2_ and CoCl_2_ (V_h_ = −40 mV, pH 5.5). (**B**): means and standard errors of the transport currents obtained from 40 oocytes, five batches. (**C–E**): Plots of fluorescence decay (F_t_/F_0_) with corresponding images of Calcein-injected oocytes (upper series: non-transfected (NT) and lower series: rDMT1 transfected) exposed to 100 μM MnCl_2_ (**C**), FeCl_2_ (**D**), and CoCl_2_ (**E**) at pH 5.5 from 3 to 10 oocytes, from 2 to 4 oocytes batches.

As a preliminary step, we have tested the ability of Calcein to detect cobalt uptake in fully grown *Xenopus laevis* oocytes. Oocytes are naturally arrested for prolonged period of time at prophase of meiosis I during which time the oocyte grows and stores macromolecular components that are necessary for future development. They exhibit different sizes that reflect different stages of growth. Fully grown oocytes, which have been used in the present paper, have a diameter of about 1.2 mm and provide a simple system for membrane transport characterization. For this purpose, we firstly needed to set up a system capable to consistently transport divalent metal ions across the plasma membrane from the extracellular milieu to the cytoplasm. We have, therefore, prepared transfected *Xenopus* oocytes by injecting them with the cRNA of the Divalent Metal ion Transporter 1 from rat (rDMT1). This membrane protein is a proton and voltage dependent transporter of divalent metal ions such as Fe^2+^ and Mn^2+^, as well as Co^2+^, Ni^2+^and Cd^2+^^29^,^30^,^31^,^32^. In mammals, it is mostly expressed in duodenum enterocytes, but it can be also found in kidney, brain, testis and placenta. By a two electrode voltage-clamp with a holding potential of −40 mV, we have recorded the currents generated by the exposure to manganese, iron and cobalt ions at pH 5.5. In non transfected (i.e., not injected with DMT1 cRNA) *Xenopus laevis* oocytes, the perfusion of ions in the bath solution did not elicit currents indicating the absence of electrogenic endogenous transporters in their plasma membrane. Conversely, in rDMT1 transfected oocytes, all the three substrates elicited, as expected^33^, inward currents in the range of −40 to −50 nA (Fig. 1A,B). As shown in Fig. 1B, iron, the physiological substrate, resulted slightly more efficiently transported than cobalt and manganese. With these experiments, we have confirmed that rDMT1 transfected oocytes, but not non-transfected ones, were able to transport iron, cobalt and manganese ions across their plasma membrane.

We have, then, filled transfected and not transfected oocytes with Calcein and monitored their fluorescence decay with an inverted fluorescence microscope. We have controlled that, before Calcein injection and at the used wavelengths, oocytes were not fluorescent (data not shown). We have also monitored the decay of the fluorescence signal in non-transfected Calcein-injected oocytes. Fluorescence decreased about 11.8% ± 2.5% in a 30 min interval (Fig. 1C–E, black squares) and, in the pH range 5.5–7.6, the decrease was pH-independent. Therefore, at our experimental conditions, only minimal photo-bleaching phenomena occurred. Similarly, non-transfected Calcein-injected oocytes which were exposed to 100 μM MnCl_2_, FeCl_2_, and CoCl_2_ (Fig. 1C–E, squares) underwent a moderate fluorescence decay not dissimilar to that occurring in the absence of the tested divalent metal ions.

Fluorescence decay was, instead, evident in transfected oocytes, i.e., in oocytes expressing rDMT1. We measured a 31.5% ± 1.1% decay for Mn^2+^ (empty triangles), a 33.2% ± 2.7% decay for Fe^2+^ (light grey triangles) and a 30.4% ± 2.4% for Co^2+^ (grey triangles). This indicates that the entry of the divalent metal ions into the cell caused the quenching of Calcein and, consequently, that Calcein can be used to monitor divalent metal ion concentration changes in the cytoplasm of *Xenopus* oocytes.

In this context, we further investigated metal-Calcein interactions by spectrofluorimetry measuring quenching in cuvettes at pH 7.6, close to the intracellular value, and at pH 5.5, value at which rDMT1 performs optimally. Values at the emission peak wavelength (i.e., 512 nm) were recorded for each concentration of Fe^2+^, Mn^2+^ and Co^2+^. The data revealed that Calcein quenching is higher for Co^2+^ and Fe^2+^, with K_0.5_ of 7.6 ± 0.7 and 5.3 ± 0.4 μM at pH 5.5 and 9 ± 3 and 0.9 ± 0.04 μM at pH 7.6. Quenching is lower for Mn^2+^ with a K_0.5_ of 53.6 ± 27 μM at pH 5.5 and of 21 ± 5.7 μM at pH 7.6. These spectrofluorometric data confirm that Calcein can be used to evaluate changes in the intracellular concentration of Mn^2+^, Fe^2+^ and Co^2+^.

### NPs cross the plasma membrane of *Xenopus laevis* oocytes

After having verified that we were able to detect an increase of divalent metal ions in the cytoplasm of *Xenopus* oocytes filled with Calcein, we have used them to reveal the possible permeation of NPs inside the cell. To this aim, we have chosen cobalt NPs in two different forms, metallic (Co^0^) and oxide (Co_3_O_4_). Both NP forms undergo dissolution^1^,^11^,^34^,^35^ releasing cobalt ions that can be detected by Calcein quenching. Therefore, we have exposed Calcein-filled oocytes to cobalt NPs and, as a control, to the corresponding ion.

**Figure 2:**
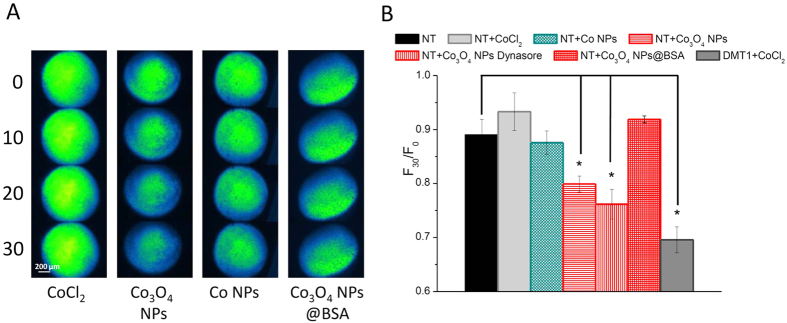
Calcein quenching in oocytes exposed to cobalt NPs. (**A**): Representative image series of non-transfected (NT) Calcein-injected oocytes exposed to CoCl_2_ or different cobalt NPs for 0, 10, 20 and 30 min. (**B**): Means of the fluorescence decay of 5 to 25 oocytes (obtained from 2 to 5 different batches). Decay is expressed as the fluorescence intensity at time 30 min over fluorescence intensity at time 0 (F_30_/F_0_). Note that quenching is statistically significant in NT oocytes exposed to bare Co_3_O_4_ NPs and in rDMT1 expressing oocytes exposed to CoCl_2_ (positive control); moreover, the endocytosis blocker Dynasore does not change the quenching effect of Co_3_O_4_ NPs. Bars are ± SE; stars indicate a statistically significant (One-way ANOVA, P < 0.05) difference with non exposed oocytes.

In oocytes from different batches, Co_3_O_4_ NPs consistently induced a quenching of Calcein fluorescence (Fig. 2). This fluorescence decrease, although lower than that occurring in rDMT1 expressing oocytes exposed to CoCl_2_ (Fig. 2B), was significantly higher than that occurring in non-transfected oocytes either exposed or not exposed to CoCl_2_. These results suggest that Co_3_O_4_ NPs interact with the plasma membrane of the oocyte, succeed in crossing it and, once in the cytoplasm, their partial dissolution causes the observed quenching activity. Indeed, cobalt ions and not NPs are able to interact with Calcein and quench its fluorescence. Co NPs, instead, did not cause a reduction of fluorescence (Fig. 2B) suggesting to be unable to pass through the plasma membrane of the oocyte. This different behavior of cobalt and cobalt oxide NPs could be ascribed to different chemical and physical characteristics of their surfaces. The importance of the surface structure of NPs in their interactions with cell membranes has been demonstrated comparing membrane penetration of two NPs that were coated with the same molecules, but arranged differently^19^. In our case, the normal spinel structure Co^2+^ Co_2_^3+^ O_2_^2−^ of Co_3_O_4_ NPs^36^ might present a surface charge distribution capable to electrostatically interact with the negative charges which are present on the plasma membrane surface; indeed, Co_3_O_4_ NPs firmly bind, through electrostatic interactions, to negatively charged biomolecules such as heparin and carboxymethylchitosan^27^,^28^. Different cationic NPs have been shown to interact with lipid bilayers and cause their disruption^37^, cationic gold NPs can enter cells by a non-endocytotic, energy-independent pathway^16^ and cationic polystyrene NPs electrostatically interact with lipid bilayers causing deformation and poration, while anionic polystyrene NPs do not^14^,^38^. In this context, Lin and Alexander-Katz^18^, with a coarse-grained simulation, have described the dynamics of cationic NP translocation through cell membranes and Nolte and colleagues^15^ have modelled the transport of spherical metal oxide NPs across a lipid bilayer.

### Protein corona impedes plasma membrane crossing

The importance of the surface characteristics of NPs in their interactions with the biological matter is well documented and is cardinal for their toxicity as well as for their use in nanomedicine. We have, therefore, modified the surface characteristics of Co_3_O_4_ NPs by letting them to adsorb bovine serum albumin (BSA). This is known to create, around the NP, a protein corona, which is capable to modify NP-membrane interaction^39^,^40^ as also suggested by computer simulations^17^. In our experiments, BSA coated Co_3_O_4_ NPs (Co_3_O_4_ NP@BSA) do not cause fluorescence quenching (Fig. 2).

These results suggest that a “protein corona” effect can prevent or significantly reduce the interactions between NPs and the oocyte membrane blocking or limiting the passage of NPs into the cytoplasm. These findings are in agreement with experiments where the interaction of cationic polystirene NPs with artificial lipid bilayers were eliminated with serum proteins^38^. Alternatively, rather than impeding NP entry into the oocytes, the protein corona could have stabilized NPs against dissolution, preventing them, once in the cytoplasm, from releasing Calcein-quenching ions. To rule out this possibility, we have performed *in vitro* experiments where NPs, which were previously exposed to BSA, were tested for their ability to quench Calcein. Since no significant differences were observed, we think that BSA treatment of NPs, although capable to generate a protein corona, was not able to prevent dissolution.

### Endocytosis does not seem to be responsible of Co_3_O_4_ NP entry

Some of our results could also be explained by endocytosis followed by NP escape from the endosomal compartment to the cytoplasm. To exclude this possibility, we have used two different approaches: in the first one, we have repeated quenching experiments on oocytes treated with Dynasore, an endocytosis inhibitor that has been used several times to block membrane recycle in *Xenopus* oocytes^41^; in the second one, we have verified whether or not NP exposure might have elicited endocytosis by optical and electron microscopy.

Dynasore is a cell‐permeable molecule that inhibits the GTPase activity of dynamin which in turn blocks dynamin-dependent endocytosis^42^. Quenching experiments were repeated in oocytes that were previously incubated for 24 h in 40 μM Dynasore. As shown in Fig. 2B, there are no significant differences in the quenching activity of Co_3_O_4_ NPs between oocytes that were treated with Dynasore and oocytes that were not.

**Figure 3:**
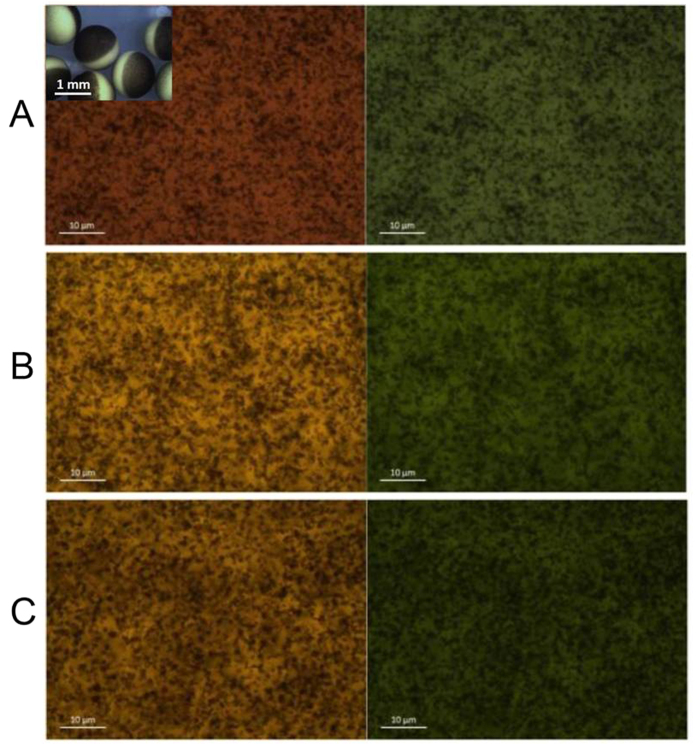
Endocytosis is not involved in NP quenching activity. In the inset, few fully grown oocytes are visible; note the presence of a pigmented pole (denominated animal pole) and of an unpigmented one (denominated vegetal pole). After fixation, a spherical cap is hand sliced with a razor blade, placed on a glass slide under a coverslip and observed with a 63X oil immersion objective from above. (**A–C**): Bright field (left) and the corresponding FITC filter (right) images of oocytes exposed to 1 mg/mL Lucifer Yellow CH. (**A**) control oocyte; (**B**) oocyte exposed to 0.1 mg/mL Co_3_O_4_ NPs and (**C**) oocyte incubated 24 h with 40 μM Dynasore and exposed to 0.1 mg/mL Co_3_O_4_ NPs. In bright field images, pigment granules, which are present in the cortex of the oocyte, are clearly visible as dark brown dots indicating that we are observing the oocyte animal pole. In the corresponding FITC filter images, no fluorescent vesicles are visible indicating no endocytotic activity.

Dynasore, however, does not halt all the endocytotic routes. Therefore, to reveal the possible formation of endocytotic vesicles after NP exposure, we have used Lucifer Yellow CH, a water-soluble and membrane-impermeable fluorescent dye; it contains a carbohydrazide (CH) group that allows it to be covalently linked to the surrounding biomolecules by aldehyde fixation. Fully grown oocytes were exposed to the tracer dye in presence and in absence of NPs for 30 min, fixed and observed under a fluorescence microscope with a 63X oil immersion objective. As shown in Fig. 3A–C, no fluorescent vesicles are visible indicating that there is minimal or no endocytosis in the tested conditions. Lucifer Yellow CH had been previously shown to be effective in tracing endocytosis occurring immediately after cortical granule exocytosis during *Xenopus* egg fertilization^43^,^44^. Likewise, treated samples were prepared also for transmission electron microscopy and, notwithstanding a careful observation of the oocyte cortex, we could not find any NP carrying endocytotic vesicle among the pigment granules and the tightly packed cortical granules which characterise the oocyte cortex (data not shown).

### No endogenous divalent metal ion transporters are present on the oocyte plasma membrane

**Figure 4:**
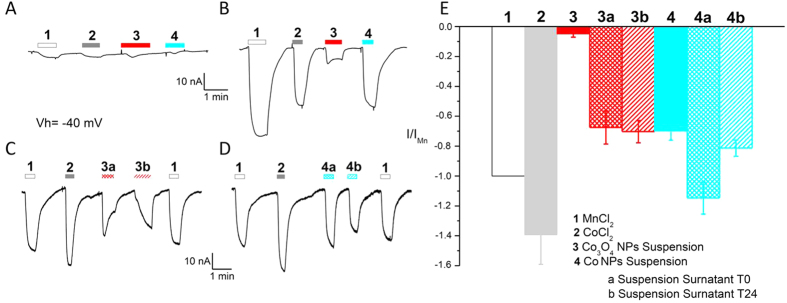
Two electrode voltage-clamp of *Xenopus* oocytes. Inward currents elicited by solutions and NP suspensions in NT (**A**) and rDMT1 transfected (**B–D**) representative oocytes. Oocytes were clamped at a holding potential of −40 mV and exposed to MnCl_2_ (1) and CoCl_2_ (2) solutions, to Co_3_O_4_ (3) and Co (4) NP suspensions and to their surnatants obtained at time 0 h (a) and 24 h (b). (**E**): Current mean values (±SE) obtained by subtracting from the current in the presence of the substrate, the current in its absence and normalizing to the Mn^2+^ current, a reference for divalent metal transporters in electrophysiological studies 6 to 12 oocytes from 4 batches.

To better characterize our system, and also to rule out possible unpredicted artefacts, we have performed further experiments which are shown in Fig. 4. We have confirmed by electrophysiology that no endogenous divalent metal ion transporters are present on the oocyte plasma membrane. Indeed, as shown in Fig. 4A, with two electrode voltage clamp no currents were recorded in the presence of CoCl_2_, MnCl_2_; similarly, no currents were recorded also in the presence of Co_3_O_4_ and Co NPs, which are known to readily dissolve releasing ions. This is in agreement with the results shown in Fig. 2 where there was not fluorescence reduction in non-transfected oocytes placed in solution containing CoCl_2_.

### Co, Co_3_O_4_ and BSA coated Co_3_O_4_ NPs release ions

Conversely, when rDMT1 transfected oocytes were tested in the presence of CoCl_2_ and MnCl_2_, inward currents were recorded (Fig. 4B), indicating an electrogenic transport of ions across the plasma membrane. Similarly, inward currents were recorded also in the presence of Co_3_O_4_ and Co NPs, indicating that both NPs, although in a different amount, were releasing ions. To confirm the release of Co ions from NPs, we have performed a set of experiments (Fig. 4C,D) exposing rDMT1 transfected oocytes to the surnatants of suspensions of Co_3_O_4_ and Co NPs and obtaining the expected inward currents. Moreover, we added BSA exposed NPs to rDMT1 expressing oocytes and, in all the tested conditions, we observed a current similar to that evoked by the same NPs that were not exposed to BSA (data not shown). These data rule out the possibility that BSA might have a role in preventing dissolution in our experiments.

### NPs do not impair oocyte plasma membrane integrity

**Figure 5:**
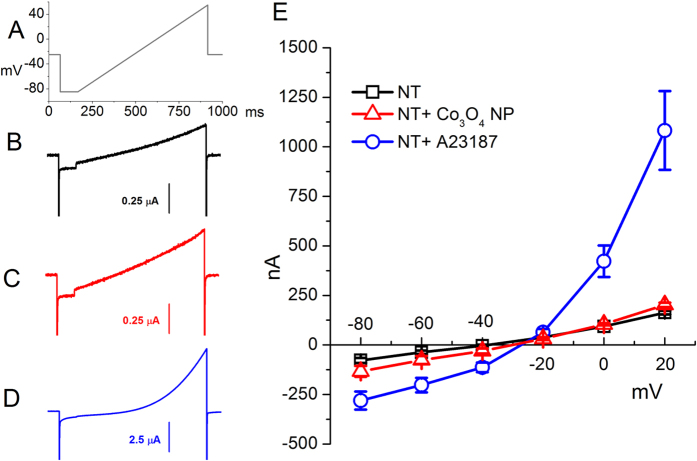
Measure of membrane resistence of *Xenopus* oocytes. Ramp (from −85 to + 55 mV) protocols (**A**) were applied in non transfected oocytes starting from the holding potential of −25 mV. Representative currents elicited by the protocol in not-exposed oocytes (black line in **B**), exposed to Co_3_O_4_ NP suspensions (red line in **C**) and perfused with ionophore A23187 (blue line in **D**). Current mean values at −80, −60, −40, −20, 0 and +20 mV (± SE) obtained from at least 12 oocytes (from 2 different batches) are plotted in (**E**).

To understand whether or not Co_3_O_4_ NPs damage oocyte plasma membrane, we have measured membrane resistence by two electrode voltage clamp. If the NPs damaged the oocyte membrane, we would expect a change in membrane permeability and the entrance of Ca^2+^ ions that, in *Xenopus* oocytes, activate Ca^2+^-gated chloride channels^45^ which, at potentials more positive than the chloride reversal potential, give rise to an outward current^46^. As shown in Fig. 5, we applyed a voltage ramp to oocytes which were previously exposed to Co_3_O_4_ NPs for 30 min and we did not observe a change in membrane resistance. Conversely, after A23187 ionophore addition^45^,^47^, a large chloride current appeared, expecially at more positive potentials, due to the activation of the Ca^2+^-gated chloride channels. Therefore, we think that NP entry does not cause injury to the plasma membrane.

In conclusion, we have demonstrated that NPs can cross cytomembranes with no evident damage to cell integrity. The canonical way of NPs to be uptaken by cells is endocytosis that makes NPs to gain access to the endosomal compartment. The NP capability to cross lipid bilayers exposes further cellular compartments to NPs. We have learned that the capability of Co_3_O_4_ NPs to cross the oocyte plasma membrane is not paralleled by that of Co NPs and that the crossing of Co_3_O_4_ NPs can be prevented by a protein corona. Moreover, we have set up a system that can be of help in evaluating the effects of different functionalizations on NP ability to cross cytomembranes. Finally, we have confirmed that Co NPs and, to a less extent, Co_3_O_4_ NPs release ions in the environment where they are present, i.e., in the extracellular solution as well as in the cytoplasm. Dissolution, indeed, is a phenomenon one should take into account not only for nanotoxicological studies, but also in nanomedicine or in food and feed fortification.

## Materials and Methods

### Solutions

ND96 solution had the following composition (in mM): NaCl 96, KCl 2, MgCl_2_ 1, HEPES 5, pH 7.6; modified Barth’s saline (MBS) solution had the following composition (in mM): NaCl 88, KCl 1, NaHCO_3_ 2.4, HEPES 15, Ca(NO_3_)_2_ 0.30, CaCl_2_ 0.41, MgSO_4_ 0.82, sodium penicillin 10 μg/mL, streptomycin sulphate 10 μg/mL, gentamycin sulphate 100 μg/mL, pH 7.6; external control solution contained (in mM): NaCl 98; MgCl_2_, 1; CaCl_2_, 1.8, HEPES or MES 5, pH 7.6 or 5.5; intracellular solution contained (in mM): KCl 130, NaCl 4, MgCl_2_ 1.6, EGTA 5, HEPES 10, glucose 5, pH 7.6. The final pH values of 5.5 or 7.6 were adjusted with HCl and NaOH.

### Oocytes collection and preparation

Oocytes were obtained from adult *Xenopus laevis* females. Animals were anaesthetised in 0.1% (w/v) MS222 (tricaine methansulfonate) solution in tap water and portions of the ovary were removed through an incision on the abdomen. The oocytes were treated with 1 mg/mL collagenase (Sigma Type IA) in ND96 calcium free for at least 1 h at 18 °C. Healthy and fully grown oocytes were selected and stored at 18 °C in MBS solution^48^. The oocytes to be transfected with the cDNA coding for rDMT1 were injected with 25 ng of cRNA in 50 nl of water, the day after the removal, using a manual microinjection system (Drummond Scientific Company, Broomall, PA) and incubated at 18 °C for 3–4 days before electrophysiological or fluorescence experiments. The experiments were carried out according to the institutional and national ethical guidelines (permit nr. 05/12).

### NP preparation

Oocytes were exposed to zerovalent (Co, 28 nm, IOLITEC, Salzstrasse 184, D-74076 Heilbronn) and oxide (Co_3_O_4_, <50 nm TEM determined, Sigma-Aldrich) cobalt NPs. 1 mg/mL Stock suspensions were prepared in deionised water. 0.1 mL of stock suspension was added to the test chamber containing 0.9 mL of external control solution (pH 7.6). Suspensions were carefully sonicated before addition to the test chamber.

For dissolution experiments, stock suspensions of Co and Co_3_O_4_ NPs were prepared to have a 10 mM concentration in terms of cobalt (i.e., 5.9 mg/10 mL for Co NPs and 8 mg/10 mL for Co_3_O_4_ NPs). Stock suspensions were sonicated for 15 min and 0.5 mL of each suspension was added to a Petri dish containing 24.5 mL of deionised water or of 1 mg/mL BSA to reach a final cobalt concentration of 200 μM.

After 1 and 24 h, 15 mL were collected from each Petri dish and centrifuged for 5 min at 8000 g at 10 °C. The surnatant was transferred in a new tube and the procedure was repeated for 4 times. Finally, 8 mL of surnatant from the last centrifugation were ultracentrifuged at 300 000 g at 4 °C for 2 h. The surnatant was collected and filtered (0.22 μm syringe filter). The resulting surnatant was diluted 1:1 in a 2X external control solution at pH 5.5 and used in electrophysiological experiments.

### Single Oocyte Fluorescence Assay (SOFA)

Untransfected oocytes and oocytes transfected with cRNA encoding rDMT1 were injected with a 50 nL drop of a 25 μM Calcein in intracellular solution. The nominal volume of a 1.2 mm diameter oocyte is 1 μL; therefore, a 50 nL injected drop will be diluted 20 times. The exact dilution factor is, however, difficult to establish, since not all the theoretical volume may be available for free diffusion^49^. Following Calcein injection, the oocytes were placed in external control solution solution at pH 5.5 or 7.6 containing or not, divalent metals at a final concentration of 0.1 mM.

For NP experiments, Co NPs and Co_3_O_4_ NPs were added to the testing solution to a final concentration of 0.1 mg/mL (pH 7.6). All experiments were carried out at room temperature. To block the endocytotic pathway, oocytes were incubated in 40 μM Dynasore (Sigma-Aldrich) for 24 h before the experiment.

Images of single oocytes were acquired every 2 min for 30 min with a fluorescence microscope (AxioVert 200, Carl Zeiss with a 4x objective, COLIBRI fluorescence filters, 470 nm excitation −515 to 565 nm emission) equipped with CCD camera (Axiocam ICM1, Carl Zeiss).

### Fluorescence and transmission electron microscopy

To assess endocytosis, oocytes were incubated for 30 min in external control solution at pH 7.6 with 1 mg/mL Lucifer Yellow CH (Sigma-Aldrich). Negative controls, oocytes exposed to 0.1 mg/mL Co_3_O_4_ NP, and oocytes pretreated with Dynasore and exposed to 0.1 mg/mL Co_3_O_4_ NPs were washed 3 times in cold (4 °C) external control solution at pH 7.6 and fixed in 4% paraformaldehyde for two days. Oocytes were washed 3 times in cold external control solution, cut in 2 halves which were placed on a slide and covered with slips. Samples were observed with a fluorescence microscope (Axiophot, Carl Zeiss) with a 63x oil ojective. Bright field and FITC filter images were taken using a CCD camera (Discovery C30, TiEsselab).

For TEM, oocytes were fixed in 4% paraformaldehide and 2% glutaraldehide in 0.1 M sodium cacodylate buffer (pH 7.4) for 24h at 4 °C and stored in 0.1 M sodium cacodylate buffer (pH 7.4) at 4 °C. Specimens were postfixed in 1% OsO_4_ in 0.1 M sodium cacodylate buffer (pH 7.4), dehydrated in ethanol and embedded in Epon-Araldite. For ultrastructural studies, thin sections were cut using a Reichert Ultracut-S-Ultratome ultramicrotome (Leica, Nussloch, Germany), collected on 300 mesh copper grids, counterstained with uranyl acetate and lead citrate and examined under a Jeol 1010 electron microscope operating at 90 KV (Jeol, Tokyo, Japan).

### Spectrofluorometry

Calcein quenching by divalent metal ions were measured in external control solution at pH 5.5 and 7.6 using a Jasco FP-750 fluorometer with excitation at 470 nm. The spectra of 2.5 μM Calcein alone or mixed with iron, manganese and cobalt ions at different concentrations were acquired in the 490 to 560 nm range. Experiments at pH 7.6 with FeCl_2_ were performed in presence of 1 mM ascorbic acid to maintain iron in a reduced form.

### Electrophysiology

The two-electrode voltage-clamp technique was performed with an Oocyte Clamp OC-725B (Warner Instruments, Hamden, CT, USA). Intracellular glass microelectrodes, filled with 3 M KCl, had tip resistances in the 0.5–4 MΩ range. Agar bridges (3% agar in 3 M KCl) connected the bath electrodes to the experimental chamber. The holding potential (V_h_) was −40 mV for the recording of transport currents and −25 mV for the measurements of membrane resistance. Currents associated to membrane transport of divalent ions were recorded in oocytes perfused with external control solution at pH 5.5 in the absence or in the presence of the indicated divalent metal ions, NPs and NP surnatants. To check for the possible presence of membrane damage, the oocyte conductances were tested applying a 1-s long protocol with a voltage ramp from −85 to + 55 mV. Positive controls of membrane conductance alteration were obtained perfusing oocytes with 10 μM ionophore A-23187 (Sigma-Aldrich). WinWCP version 4.4.6 (J. Dempster, University of Strathclyde, UK) or Clampex 10.2 (Molecular Devices, Sunnyvale, CA, USA, www.moleculardevices.com) were used to run the experiments.

### Data analysis

Data were analysed using Clampfit 10.2 software (Molecular Devices, Sunnyvale, CA, USA, www.moleculardevices.com) while OriginPro 8.0 (OriginLab Corp., Northampton, MA, USA, www.originlab.com) was used for statistics and figure preparation. Transport currents were determined by subtracting the records in the absence of a substrate from the corresponding ones in its presence. Fluorescence decay images were analysed with ImageJ (Rasband, W.S., ImageJ, U. S. National Institutes of Health, Bethesda, Maryland, USA, http://imagej.nih.gov/ij/, 1997–2015). For F_t_/F_0_ quantification, the fluorescence intensity at time 0 (F_0_) and at subsequent times (F_t_) was calculated in the entire area of the oocyte.

## Additional Information

**How to cite this article**: Bossi, E. *et al.* Cobalt oxide nanoparticles can enter inside the cells by crossing plasma membranes. *Sci. Rep.* **6**, 22254; doi: 10.1038/srep22254 (2016).
